# Supplementary material for: Development of Molecular-Based Species Identification and Optimization of Reaction Conditions for Molecular Diagnosis of Three Major Asian Planthoppers (Hemiptera: Delphacidae)
Source: Insects. 2023 Jan 25;14(2):124. doi: 10.3390/insects14020124 (PMC9962309; doi:10.3390/insects14020124)
Supplement: Supplementary file 1 [file insects-14-00124-s001.zip › insects-2085578-supplementary.docx]

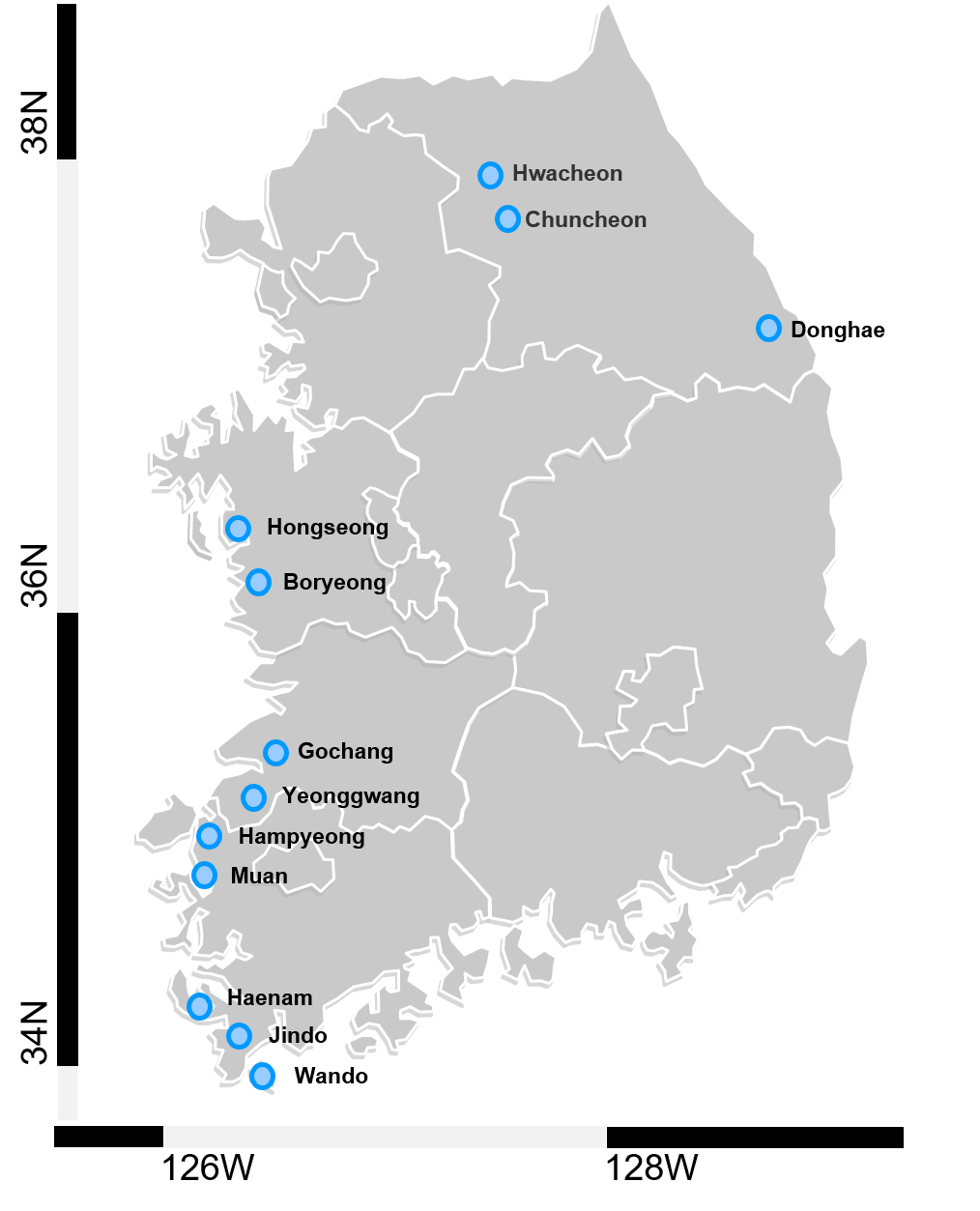


**Figure S1**: Sample collection regions from different localities in South Korea. The blue-colored round shape indicates the planthopper (PH). Regional indication: CC, Chuncheon; HC, Hwacheon; DH, Donghae; HS, Hongseong; BR, Boryeong; GC, Gochang; YG, Yeonggwang; HP, Hampyeong; MA, Muan; HN, Haenam; JD, Jindo; WD, Wando.


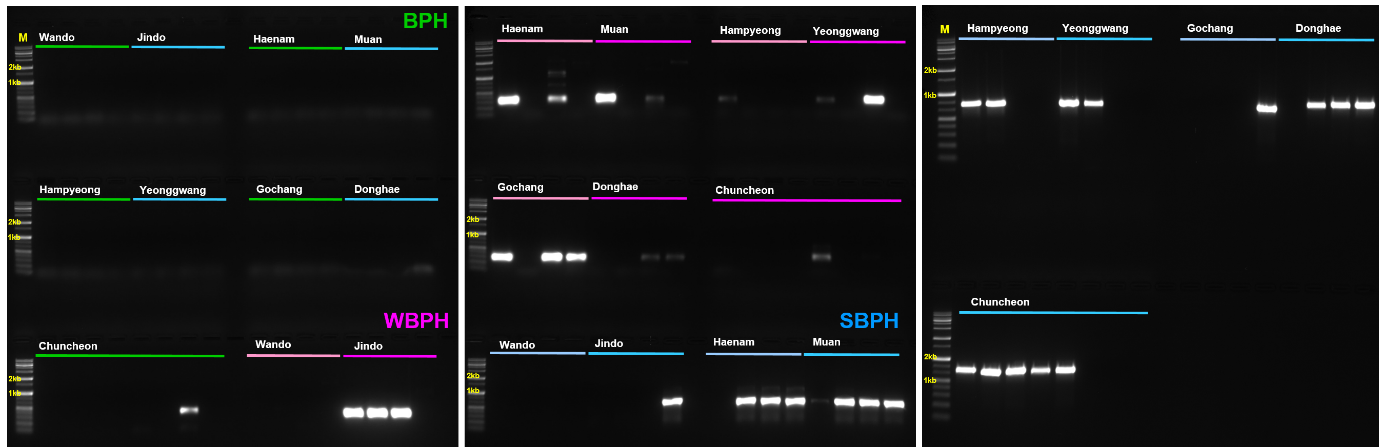


**Figure S2**. Validation/field application with randomly collected insects from nine different locations (Wando, Jindo, Haenam, Muan, Hampyeong, Yeonggwang, Goechang-gun, Donghae-si, and Chuncheon-si). NTC: no template control, BPH: brown plant hopper, *Nilaparvata lugens*, SBPH: small brown planthopper, *Laodelphax striatellus*, WBPH: white-backed planthopper, *Sogatella furcifera.*
